# Graphene for Electronics

**DOI:** 10.3390/nano12244359

**Published:** 2022-12-07

**Authors:** Eugene Kogan

**Affiliations:** Department of Physics, Bar-Ilan University, Ramat-Gan 52900, Israel; eugene.kogan@biu.ac.il

Graphene is an allotrope of carbon consisting of a single layer of atoms arranged in a two-dimensional (2D) honeycomb lattice. Graphene’s unique properties of thinness and conductivity have led to global research into its applications as a semiconductor. With the ability to conduct electricity well at room temperature, graphene semiconductors could easily be implemented into the existing semiconductor technologies and, in some cases, successfully compete with the traditional ones, such as silicon. Research has already shown that graphene chips are much faster than existing ones made from silicon. The world’s smallest transistor was manufactured using graphene. Flexible, wearable electronics may take advantage of graphene’s mechanical properties, as well as its conductivity, to create bendable touch screens for phones and tablets, for example.

On the other hand, the physics of graphene and graphene-based systems has inspired the application (and development) of many advanced theoretical methods, including those outside the scope of traditional condensed matter physics. Graphene thus turned into the favorite benchmark of theorists. Fundamental studies go hand in hand with the applied ones and, in some cases, the former even opened doors to possible applications.

Graphene has already led to substantial progress in the development of the current electronic systems due to its unique electronic and thermal properties, including its high conductivity, quantum Hall effect, Dirac fermions, high Seebeck coefficient and thermoelectric effects. It paves the way for advanced biomedical engineering, reliable human therapy, and environmental protection. This suggests substantial improvements in current electronic technologies and applications in healthcare systems.

This Special Issue of Nanomaterials covers recent studies, both theoretical and experimental, that advance our understanding of graphene and may be relevant to graphene electronics. With the growing number of flexible electronics applications, environmentally friendly ways of mass-producing graphene electronics are required. Kralj and coworkers [1] present a scalable mechanochemical route for the exfoliation of graphite in a planetary ball mill with melamine to form melamine-intercalated graphene nanosheets.

Field-effect transistors have attracted significant attention in chemical sensing and clinical diagnosis, due to their high sensitivity and label-free operation. Huang and coworkers [2] present the study of a scalable photolithographic process of fabrication of the graphene-based ion-sensitive field-effect transistor (ISFET) arrays.

The study of electronic transport in the lowest Landau level of disordered graphene sheets placed in a homogeneous perpendicular magnetic field, a long-standing and cumbersome problem which defied a conclusive solution for several years, is presented in the paper by Sinner and Tkachov [3].

The paper by Berman et al. [4] contains theoretical analysis of Bose-Einstein condensation and superfluidity of dipolar excitons, formed by electron-hole pairs in spatially separated gapped hexagonal layers.

The electrical properties of polycrystalline graphene grown by chemical vapor deposition are determined by grain-related parameters, such as average grain size, single-crystalline grain sheet resistance, and grain boundary resistivity. Park et al. [5] have observed that the material property, graphene sheet resistance, could depend on the device dimension and developed an analytical resistance model based on the cumulative distribution function of the gamma distribution, explaining the effect of the grain boundary density and distribution in the graphene channel.

Electric devices have evolved to become smaller, more multifunctional, and increasingly integrated. When the total volume of a device is reduced, insufficient heat dissipation may result in device failure. A microfluidic channel with a graphene solution may replace solid conductors for simultaneously supplying energy and dissipating heat in a light emitting diode (LED). Chung et al. [6] designed, using a graphene solution, an automated recycling system that reduces the necessity of the manual operation of the device.

Silkin and coworkers [7] present a detailed first-principles investigation of the response of a free-standing graphene sheet to an external perpendicular static electric field.

The tunneling of electrons and holes in quantum structures plays a crucial role in studying the transport properties of materials and the related devices. A new two-dimensional Dirac material, 8-Pmmn borophene, hosts tilted Dirac cone and chiral, and anisotropic massless Dirac fermions. Kong et al. [8] adopted the transfer matrix method to investigate the Klein tunneling of massless fermions across the smooth NP junctions and NPN junctions of 8-Pmmn borophene.

The process of formation of carbon nanoscrolls with non-uniform curvatures is worthy of a detailed investigation. Lin et al. [9] present the first-principles method suitable for studying the combined effects due to the finite-size confinement, the edge-dependent interactions, the interlayer atomic interactions, the mechanical strains, and the magnetic configurations.

In the paper by Krasovskii [10], angle-resolved photoemission from monolayer and bilayer graphene is studied based on an ab initio one-step theory.

In the paper by Do [11] and coworkers, by introducing a generalized quantum-kinetic model which is coupled self-consistently with Maxwell and Boltzmann transport equations, the authors elucidate the significance of using input from first-principles band-structure computations for an accurate description of ultra-fast dephasing and the scattering dynamics of electrons in graphene.

In summary, this Special Issue presents several examples of the latest advancements on graphene science. We hope the readers will enjoy these articles and find them useful for their research.
